# Cardiac Magnetic Resonance and Ventricular Arrhythmia Risk Assessment in Chronic Ischemic Cardiomyopathy: An Unmet Need?

**DOI:** 10.31083/j.rcm2307246

**Published:** 2022-06-28

**Authors:** Beatriz Jáuregui, Naiara Calvo, Teresa Olóriz, Carlos López-Perales, Antonio Asso

**Affiliations:** ^1^Arrhytmia Section, Cardiology Department, Miguel Servet University Hospital, 50009 Zaragoza, Spain

**Keywords:** cardiac magnetic resonance, ischemic cardiomyopathy, left ventricular ejection fraction, myocardial infarction, arrhythmogenic substrate, ventricular tachycardia, sudden cardiac death

## Abstract

Ischemic cardiomyopathy (ICM) constitutes a major public health issue, directly involved in the prevalence and incidence of heart failure, ventricular arrhythmias (VA) and sudden cardiac death (SCD). Severe impairment of left ventricular ejection fraction (LVEF) is considered a high-risk marker for SCD, conditioning the criteria that determine an implantable cardiac defibrillator (ICD) placement in primary prevention according to current clinical guidelines. However, its sensitivity and specificity values for the prediction of SCD in ICM may not be highest. Myocardial characterization using cardiac magnetic resonance with late gadolinium enhancement (CMR-LGE) sequences has made it possible to answer clinically relevant questions that are currently not assessable with LVEF alone. There is growing scientific evidence in favor of the relationship between fibrosis evaluated with CMR and the appearance of VA/SCD in patients with ICM. This evidence should make us contemplate a more realistic clinical value of LVEF in our daily clinical decision-making.

## 1. Introduction

Ischemic cardiomyopathy (ICM) and its consequences are estimated to be the cause of at least 80% of sudden cardiac deaths (SCDs) (around 240,000–280,000 per year) in Western countries [1]. From autopsy data, around 30% of all the ICM-related SCDs lack acute coronary findings, but are instead linked to the presence of an old ischemic myocardial scar [2]. The biological processes of myocardial healing, left ventricular (LV) remodeling and scar changes involve the development of a potential arrhythmogenic substrate capable of causing reentrant ventricular arrhythmias (VA), the main cause of arrhythmic SCD in chronic ICM [3].

Ventricular fibrillation (VF) constitutes the final VA leading to arrhythmic SCD. Polymorphic ventricular tachycardias (VTs) are usually related to acute ischemia and can quickly degenerate into VF [4]. Monomorphic VTs (MVTs) are due to anatomical/functional reentry related to the presence of myocardial scar/fibrosis, and may degenerate into VF owing to primary rhythm disorganization, hemodynamic instability and secondary global ischemia. In patients with chronic ICM, VAs causing SCD are mostly reentrant MVTs [5] that require the presence of a histologic substrate for reentry. However, several external triggers may also be required for their initiation, such as neurohormonal alterations, mechanical factors (e.g., myocardial stretching in conditions of volume/pressure overload), and ionic alterations [6, 7].

Severe LV systolic dysfunction is considered a high-risk marker for SCD [8]. In current clinical practice guidelines [9, 10], the presence of a left ventricular ejection fraction (LVEF) <35% in chronic ICM is the criterion that determines an implantable cardiac defibrillator (ICD) placement in primary prevention of SCD [11, 12]. However, only 20–30% of the patients included in randomized clinical trials on primary prevention receive appropriate ICD shocks after 4 years of follow-up, indicating limited positive predictive value of LVEF as a risk marker for SCD [11, 12]. In addition, approximately 65% ​​of patients with SCD have normal or only mildly depressed LV systolic function (i.e., LVEF between 35% and 50%) [13, 14, 15]. Therefore, severe LV dysfunction alone is a not sufficiently specific marker for SCD, although it could be useful when used with other predictors or as part of a multivariable risk score including various clinical parameters, such as age, New York Heart Association (NYHA) functional class, renal function, QRS width, coexistence of atrial fibrillation, VT inducibility during electrophysiological studies (EPS), myocardial hypertrophy, heart rate variability, etc. [16, 17, 18, 19]. However, such scoring algorithms have not yet been validated in large prospective series. Besides, these markers are not very specific when it comes to discriminating the risk of SCD versus the risk of non-sudden death [16, 17, 20, 21].

## 2. Conventional Arrhythmia Risk Assessment in Ischemic Cardiomyopathy

### 2.1 Left Ventricular Ejection Fraction

Mortality and SCD risk increase when LVEF decreases below 50% [22, 23], and particularly when it becomes <40% [24]. On the other hand, due to the improvement in reperfusion therapies, there are relatively few patients with very low LVEF after an acute coronary event [25, 26, 27]. However, there is no evidence of a cause-effect relationship between an abnormal LVEF and the occurrence of SCD. In fact, although the absolute risk of SCD is higher in patients with lower LVEF, the total number of SCDs is higher in patients with higher LVEF [28]. In addition, current LVEF values used to define high-risk populations (typically <35%) have poor sensitivity, failing to identify up to 50% of patients at risk for SCD [29].

Current evidence for the choice of LVEF as the main criterion for ICD implantation in primary prevention in patients with ICM is based on the classical trials MADIT-I and II [11, 30], CABG-PATCH [31], MUSTT [32], DINAMIT [33], and SCD-HeFT [12]. Although in the latest clinical practice guidelines the presence of LVEF <35% remains the criterion that currently determines implantation [11, 12], it has not been shown to be a parameter either sensitive or specific for the prediction of SCD in ICM [34, 35, 36, 37, 38, 39]. In the recent PRESERVE EF study [40] it was found that 9 of 575 patients (1.6%) with ICM and LVEF >40% (mean 50.8%) had appropriate ICD therapies due to sustained VA after a mean clinical follow-up of 32 months. These patients, who did not have a standard indication for ICD implantation in primary prevention, had been indicated for ICD because they had some non-invasive additional risk factor (see below) and were inducible during EPS.

### 2.2 Other Parameters

Traditionally, other clinical variables apart from LVEF have been tested in order to improve the risk stratification of VA/SCD in patients with ICM. Some of these variables have been early detection of potential triggers (premature ventricular complexes and non-sustained VTs), presence of late potentials on the signal-averaged ECG, T wave alternans, inducibility during EPS, analysis of autonomic functional markers, or the use of multivariable clinical models. The available evidence has confirmed that none of them was sufficiently reliable for arrhythmic risk stratification [19, 41, 42, 43, 44, 45, 46, 47, 48, 49]. Therefore, their use cannot be recommended for making relevant clinical decisions.

## 3. Current Status and Role of Cardiac Magnetic Resonance for Arrhythmia Risk Assessment in Ischemic Cardiomyopathy

### 3.1 Role of Cardiac Magnetic Resonance to Calculate Left Ventricular Ejection Fraction

As previously mentioned, LVEF is a suboptimal risk marker for VA and SCD, and yet it constitutes the main criterion on which current clinical practice guidelines base the indication for ICD implantation in primary prevention of SCD [11, 12]. LVEF has been classically obtained using echocardiography, which has shown to generally overestimate LVEF by 3–7% compared to cardiac magnetic resonance (CMR), a more reproducible technique [50]. These differences might justify a better risk stratification using CMR [51, 52], despite no published ICD trial has included the use of CMR-derived LVEF. On the other hand, there is evidence [53] suggesting that the use of a weighted CMR score after a ST-segment–elevation myocardial infarction, including CMR-LVEF and other variables (myocardial salvage index, microvascular obstruction, and myocardial hemorrhage), was independently associated with major adverse cardiovascular events, and may provide incremental prognostic stratification as compared with GRACE score and echo-LVEF [53].

### 3.2 Role of Cardiac Magnetic Resonance to Identify the Arrhythmogenic Substrate

Far beyond LVEF, other clinical predictors for arrhythmia events may be found; for example, in a recent meta-analysis including 17 studies with LVEF values ranging from 25 to 59% [54], the presence of a coronary chronic total occlusion (CTO), particularly when affecting the infarct-related artery, was associated with a 1.68-fold increase in the occurrence of VT/VF or appropriate ICD therapy. Aiming to non-invasively characterize the post-infarction myocardial injury, cardiac magnetic resonance with late gadolinium enhancement (CMR-LGE) has made it possible to answer clinically relevant questions that are currently not assessable with LVEF alone or with the use of other non-invasive imaging modalities. In fact, the diagnostic and prognostic role of CMR in ICM has already been broadly recognized in more recent articles, guidelines and consensus documents [55, 56, 57, 58]. Numerous studies have shown a link between the presence of macroscopic fibrosis evaluated with CMR and the appearance of VA in patients with ICM. In a recent meta-analysis by Disertori *et al*. [59] including 2850 patients from 19 different studies, patients without LGE had an annual arrhythmia/SCD event rate of 1.7%, compared with an event rate of 8.6% per year in patients with LGE. Moreover, 100% of those with events had macroscopic fibrosis on CMR-LGE [59, 60]. This has been supported in modern, large prospective series of patients with ICM and non-ischemic etiologies [61].

The ability of CMR-LGE to detect myocardial fibrosis has been supported by histological correlation studies [62, 63]. The presence of slow and heterogeneous electrical conduction associated with fibrosis may favor reentry, increasing vulnerability to VT/VF onset [6, 64, 65, 66]. Areas with different levels of fibrosis coexist in the gray zone, heterogeneous tissue, or border zone (BZ), resulting in simultaneous regions of viable and non-viable myocardium, which can be identified with CMR-LGE [64]. In addition, heterogeneous tissue channels (HTC), or border zone channels (BZC), have been correlated with functional, slow conduction channels identifiable by endocardial voltage maps during electroanatomical mapping (EAM) [67, 68, 69, 70], as well as with the critical isthmuses of VT reentry circuits [71].

Despite the evidence that size and heterogeneity of the myocardial scar could be predictors of VA and mortality, it remains unknown whether CMR, beyond the use of LVEF, could facilitate the clinical decision-making in relation to arrhythmia risk stratification. In this sense, a previous prospective study by Klem *et al*. [72], based on a cohort of ischemic patients with a wide range of LVEF values, analyzed the risk of arrhythmic events: Patients with LVEF >30% and fibrosis >5% of total myocardial mass had a higher VA risk than those patients with LVEF >30% and small scars (<5%), but similar to that of patients with LVEF <30%. Similarly, those patients with LVEF <30% and fibrosis <5% had a risk of arrhythmic events similar to that of patients with LVEF >30%. More recently [73], the presence of myocardial fibrosis and the mass of heterogeneous tissue (a.k.a. BZ), both analyzed with CMR-LGE, were evaluated in relation to the occurrence of SCD and the composite event of SCD or VA. Of 947 patients analyzed retrospectively [73], there were 29 cases of SCD (2.96%) and 80 cases of SCD/VA (8.17%) after a median follow-up of 5.82 years. The visual presence of myocardial fibrosis or heterogeneous tissue on CMR was strongly associated with the appearance of SCD and the composite event. In contrast, the associations between LVEF <35% and the development of SCD, or the composite SCD/VA, were much weaker in a competing risk analysis [73]. In addition, all patients having events (SCD/VA) showed evident fibrosis on CMR. Furthermore, the cut-off point of LVEF <35% was very weakly associated with both the occurrence of SCD or the composite SCD/VA [73]. Additionally, from all the scar variables that can be assessed with CMR, the amount (mass) of BZ and, particularly, the amount of BZ mass being part of tissue corridors (BZC) appear to be the most powerful variables associated with the development of reentrant VA [74, 75].

Including CMR-derived variables, the currently ongoing European PROFID project will aim to develop a clinical prediction model for SCD in ICM in order to improve the selection of ICD candidates. Two randomized trials will validate the usefulness of this prediction model according to the LVEF (PROFID-Preserved, NCT04540289; and PROFID-Reduced, NCT04540354). The preliminary results presented in EHRA Congress 2022 indicate a potentially relevant role of CMR-LGE; particularly regarding the extent of scar and BZ. Therefore, un updated model including these variables is to be expected.

Apart from evaluating macroscopic fibrosis using LGE, CMR is also capable of assessing diffuse interstitial myocardial fibrosis, which is related to adverse remodeling in patients with ICM [76, 77]. This is usually performed using T1-mapping techniques. Diffuse fibrosis has been shown to be an independent predictor of VA [78]. However, it should be remarked that T1-mapping is limited to just a single slice of myocardium, assuming that the diffuse fibrosis affects uniformly the remote myocardium. Therefore, more studies would be required to explore the possibilities of this method.

### 3.3 Role of Cardiac Magnetic Resonance during Ventricular Tachycardia Substrate Ablation Procedures

In addition to the aforementioned, it has recently been shown that the QRS morphology of potential inducible VTs and the location of the responsible circuit in each case can be accurately predicted using computer simulation models trained with CMR-LGE images from infarcted pigs [79]. This may suggest that our current post-processing methods for BZ analysis could eventually be complemented with even more sophisticated tools (computer models, machine learning, etc.) to identify reentrant circuits through CMR findings. Other studies have strongly supported the usefulness of CMR in identifying and characterizing the arrhythmogenic substrate in secondary prevention. Integrated 3D reconstructions of the scar obtained from CMR images are capable of accurately depicting not only the location of the arrhythmogenic substrate, but also its structure and the presence of BZC, which constitute the therapeutic target during substrate ablation procedures [80, 81, 82]. These studies have demonstrated improved clinical outcomes and better procedural efficiency when taking advantage of the CMR-derived data on the arrhythmogenic substrate.

## 4. Conclusions

There is growing scientific evidence in favor of the relationship between CMR-LGE findings and the appearance of VA/SCD, particularly in ICM but also in non-ischemic cardiomyopathies. This evidence should make us contemplate a more realistic clinical value of LVEF in our daily clinical decision-making. Nevertheless, additional studies are still required. Standardization of image acquisition methods and measurements, as well as analysis of the diagnostic performance of each evaluable scar-derived parameter are issues to be resolved. Furthermore, the dynamic nature of scar remodeling makes it difficult to set an appropriate time point for CMR acquisition [83]. Finally, there is an urgent need for reliable post-processing tools allowing reproducible analysis of raw CMR images. Undoubtedly, the key for diagnosis and prognosis in our patients lies within them.
